# Studying the Deep Evolution of Viruses in the Era of AI Structure Prediction

**DOI:** 10.1146/annurev-virology-100424-122154

**Published:** 2026-09-25

**Authors:** Spyros Lytras, Mahan Ghafari, Joe Grove

**Affiliations:** 1 Antigen Evolution and Design Lab, Department of Structural Biology and Chemistry, Institut Pasteur, Paris, France; 2 Department of Biology, University of Oxford, Oxford, UK; 3 Pandemic Sciences Institute, University of Oxford, Oxford, UK; 4 Lincoln College, University of Oxford, Oxford, UK; 5 MRC-University of Glasgow Centre for Virus Research, Glasgow, UK

**Keywords:** Virus Evolution, Virus Discovery, Virus Taxonomy, Protein Structure Prediction, AlphaFold, Structural Phylogenetics

## Abstract

High mutation rates erode viral sequence similarity, obscuring deep evolutionary history. While protein structure is far more conserved than sequence, its use in evolutionary studies has historically been bottlenecked by experimental determination. The recent revolution in AI-structure prediction has fundamentally changed this, enabling the rapid generation of millions of viral protein structures. This review examines the impact of these technologies on our understanding of deep viral evolution. We describe the strengths and limitations of protein structure prediction and consider the questions it can be used to address: illuminating viral ‘dark matter’ in metagenomic datasets, resolving high-level taxonomy for ‘orphan’ lineages, and inferring function for divergent proteins. Furthermore, we assess the emerging field of structural phylogenetics, exploring the theoretical and practical challenges of integrating structure and sequence to reconstruct ancient evolutionary events. We conclude that despite remaining challenges, systematic structure prediction will extend our exploration of deep evolution across the Virosphere.

## 1 Introduction

The Virosphere - the sum total of all viruses on Earth - presents a convoluted web of evolutionary histories, interwoven with that of their hosts. Any given virus is composed of a collection of proteins and genetic elements, each with their own origins and journeys through evolutionary time. Concurrent processes drive this complexity: short life cycles and error-prone genome replication lead to rapid evolutionary rates and extraordinary genetic diversity, while recombination and horizontal gene transfer produce mosaic genomes (1–5). At the same time, host-virus “arms races” leave long-lasting signatures on both viral and host genomes (6, 7). These forces act on existing features and functions, but also drive the *de novo* emergence of novel genes (8–10).

Constant genetic turnover allows recent evolution to be observed at high resolution (11). Concerted sequencing efforts can track adaptation and immune-escape of seasonal or emergent pathogens in, near-enough, real-time (12–14). However, these approaches are limited for reconstructing very deep evolutionary histories, such as the origins of viral families. These challenges are pronounced for RNA viruses, where rapid mutation erodes sequence similarity, pushing homologous relationships toward the “twilight zone” (<25% amino acid identity (15)). Here, sequence alignments become unreliable and phylogenetic signal degrades. Consequently, the huge advances in our ability to survey the Virosphere through metagenomics - seen over the last decade (16) - are disconnected from our ability to navigate this diversity and reconstruct the events that gave rise to it.

In the absence of a robust phylogenetic signal, researchers often turn to alternative evidence to infer deep relationships. These include ancient DNA recovered from archaeological human remains (17) or archival pathology samples (18); biogeographic patterns (19); paleovirological records in the form of endogenous viral elements (20, 21); and assumptions about long-term host–virus co-evolution (22). While efforts exist to mechanistically model sequence dynamics over long periods (23), they remain constrained by the lack of nucleotide or amino acid homology.

Protein structure has long been known to reveal even deeper evolutionary relationships than sequence analysis. To retain function, the fold of a protein - the path the polypeptide chain traces through three-dimensional space - is far more conserved than the underlying sequence. Furthermore, protein folds typically exhibit convoluted topologies, which contain high information content that can be used to demonstrate homology even without sequence similarity, extending detection far beyond traditional phylogenetics (24, 25). This principle was apparent in the earliest studies: the first two experimentally solved protein structures, myoglobin and hemoglobin, share a conserved globin-fold whilst exhibiting little sequence homology (26–28). However, despite subsequent advances in structural biology - including the “resolution revolution” in cryogenic electron microscopy (29) - the experimental determination of protein structure remains a technically challenging, time-consuming and costly endeavour. Indeed, in 2024, the Protein Data Bank grew ~15,000 new entries (30), whereas NCBI received >1 billion whole genome nucleotide sequences (31). This fundamental difference in scale has limited the utility of structural homology, over sequence homology, for systematic studies of deep evolution.

We are, however, experiencing a new revolution in structural biology. AI breakthroughs in natural language processing (32) have been repurposed for the language of proteins. AI-based protein structure prediction methods, most notably AlphaFold (33, 34), now make it possible to generate accurate structural models in seconds to minutes, using widely available cloud computing (35). This technology scales with sequencing approaches, enabling systematic, structure-based exploration of evolutionary histories (36). It is for viruses - with their extraordinary diversity - that the structure prediction revolution will arguably have the greatest impact.

## 2 Exploring protein diversity with AI structure prediction

Computational protein structure prediction has been charted for over 30 years by the biannual CASP (Critical Assessment of Structure Prediction) competition, in which computational approaches are benchmarked against a selection of target sequences for which experimental structures have been solved but not yet released (37–39). In 2020 (CASP14) AlphaFold2 set a new state of the art in prediction, achieving near-experimental accuracy (33). Whether the protein folding problem has truly been solved remains a contentious topic. Nonetheless, with 1 Nobel Prize and more than 48,000 citations at the time of writing, AlphaFold2 has had a transformative impact on molecular biology. Importantly, it performs well against viral proteins, including novel viral folds and targets that had previously been recalcitrant to lab-based characterisation (40–44).

### 2.1 Co-evolution and confidence

While a deep technical understanding of structure prediction is not required for the study of viral evolution, an orientation on its strengths and limitations is beneficial. AlphaFold does not explicitly simulate the physical laws of protein folding; instead, it has learned the relationship between evolution and structure. Residues distant in sequence can co-evolve (e.g., maintaining charge balance) in a manner that reflects their proximity in three-dimensional structure space (45, 46). These relationships can be identified in multiple sequence alignments (MSAs) containing many homologous protein sequences and used to inform structure prediction (47–50). For example, Marks et al. demonstrated that co-evolutionary coupling is sufficiently well correlated with 3D distance to predict residue-residue contact maps and to perform accurate *de novo* structure prediction (47). AlphaFold2 uses a transformer-based deep neural network to reconcile these co-evolutionary signals with geometric constraints to produce highly accurate predicted structures (33). While recent proprietary and open-source methods continue to improve accuracy, particularly for protein complexes (23, 41–44), MSA analysis remains central to high-performing approaches.

Reliance on MSAs, however, represents an Achilles’ heel. Sequences with few homologs produce shallow MSAs (<30 sequences), resulting in poor predictions (33, 51). This is common for highly divergent viral sequences discovered through metagenomics (24). Here, MSA-free approaches using protein language models (pLMs), such as ESMFold (52), offer a solution. Trained on hundreds of millions of sequences, pLMs have “learned” the complex pairwise relationships between residues in a protein (52–55). Their ability to infer residue-residue contact maps is an emergent feature of pLMs, and can be used to predict protein structure using a single sequence, rather than an MSA (56). While generally less accurate than MSA-based methods, pLMs can “rescue” the folds of sequences with few homologs and can be valuable for surveying divergent viral sequences (2, 24, 43, 57). Moreover, structural inference with pLMs is more than 10-fold faster than MSA-based approaches (52); making them particularly well suited to large-scale prediction tasks.

Regardless of the method, all structure prediction approaches provide confidence metrics that are well-correlated with accuracy (33, 52, 58, 59). Predicted local distance difference test (pLDDT) is a per-residue score (ranging 0-100) capturing local confidence, pertaining to side chain position and secondary structure (60). AI-predicted protein structures are commonly presented colour-coded by pLDDT, providing an instant readout on quality, with shades of blue and green indicating high confidence. Albeit, that low pLDDT can indicate intrinsically disordered regions, rather than prediction failure (61, 62). The predicted template modelling (pTM) score (ranging from 0 to 1) measures global fold quality, and represents the estimated similarity between a predicted structure superposed on a hypothetical matched “ground truth” structure (63). Evaluating these metrics is a prerequisite for using models in downstream evolutionary inference.

### 2.2 Scaling up AI structure prediction to the Virosphere

**Figure 1:**
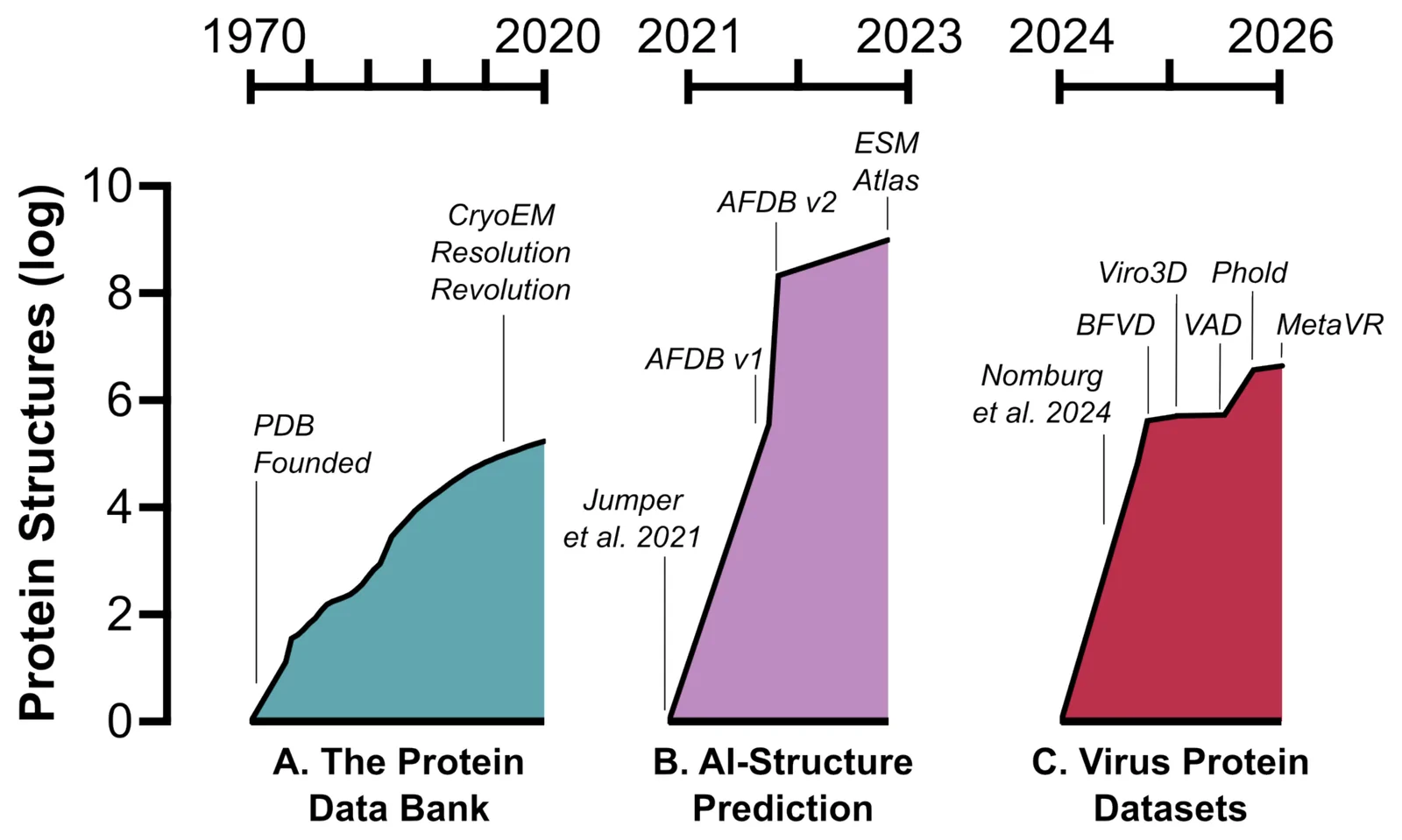
The growth of protein structure databases. **A)** Experimental structural biology (X-ray crystallography, NMR and, latterly, CryoEM) enabled the hard-fought accumulation of protein structures in the PDB. **B)** AI-structure prediction enabled the generation of ~1 billion protein structures in approximately two years, but largely excluded viral proteins. **C)** 2024-2025 saw the delivery of ~4.4 million viral protein structures across multiple datasets. AFDB (AlphaFold Database), BFVD (Big Fantastic Virus Database), VAD (Virus AlphaFold Database).

Whilst the Protein Data Bank of experimental structures (64) provided essential training data for current AI approaches, it does not sufficiently sample protein diversity for systematic homology-based exploration of viral evolution (Figure 1A). For example, of ~34K viral PDB structures ~25% are from just two viruses: SARS-CoV-2 and HIV-1. The scalability of AI-inference permits the rapid generation of databases that vastly increase structural coverage. The AlphaFold Protein Structure Database (AFDB) - a collaboration between Google DeepMind and EMBL-EBI - contains >200 million structures, including proteomes of 16 model organisms and 30 WHO priority pathogens (65). However, at the time of its launch in 2021, the AFDB systematically excluded viral proteins. Meta’s ESM Metagenomic Atlas, generated using ESMFold (52), contains >772 million proteins from the Mgnify database (66), includes some viral proteins, but without annotation or taxonomic labels. Consequently, these efforts (Figure 1B) - performed using the significant computing power of big tech - did not build the resources required for virology.

This gap is, however, being filled. Over the space of one year, community-led efforts have delivered ~4.4 million viral protein structure predictions, providing the raw materials for structure-guided exploration of viral diversity (Figure 1C). At the time of writing there are eight independent, large-scale, virus-focussed datasets, summarised in Table 1 (8, 43, 67–72). While an in-depth comparison of these rapidly evolving resources is beyond the scope of this review, several themes have emerged. First, viral protein structures can be difficult to predict, often due to insufficient MSA depth, resulting in a significant minority of low-confidence and inaccurate structures. However, these limitations can be overcome. MSA enrichment through non-standard or custom sequence databases can rescue-low confidence predictions. For example, Kim et al. (67) supplemented standard sequence databases with the Logan massive sequence assembly for BFVD (73), while Phold drew from a custom database of phage proteins (72). Alternatively, pLM-based prediction methods are MSA-independent; ESMFold outperformed AlphaFold2/ColabFold on ~10% of targets in Viro3D and Phold (43, 72). Finally, the Viral AlphaFold Database (VAD) demonstrated that modeling homodimers can rescue low-confidence monomeric folds, showing that co-evolutionary signals - used to inform prediction - propagate within oligomeric arrangements (68).

A final consideration is the trade-off between depth of sequence coverage and breadth of structural diversity. Thus far, databases have either focussed on depth of sequence coverage within a particular subset of the Virosphere (e.g., ICTV-classified vertebrate and invertebrate viruses by Viro3D) or have prioritised sampling structural diversity through clustering of targets based on sequence identity (e.g., BFVD). While the latter structure-focused approach is ideal for identifying distant deep homology, the former sequence-focused strategy remains necessary to fill in the intervening evolutionary trajectories.

### 2.3 Navigating structural diversity

Exploiting structural similarity to traverse large evolutionary distances requires sensitive tools for structural alignment. The computational comparison of tertiary structures has been explored for over 50 years (74), with methods progressively accounting for indels, divergence between compared structures (75), evaluating pairwise homology (76, 77), and considering structural flexibility (78, 79), with many methods being adapted for multiple structure comparisons (80–83). Until recently, the most widely used and sensitive approaches (e.g., DALI, CE and TM-align) (77, 84, 85) performed pairwise 3D alignment and similarity scoring. While excellent for detecting homology, these methods are computationally expensive, having been developed when the principal bottleneck was the availability of experimental structures. The sudden arrival of ~1 billion predicted structures via AFDB and the ESM Metagenomic Atlas demanded new approaches built for scale.

The inefficiency of comparing structures stems from the high amount of information encoded in a three-dimensional molecule. Hence, instead of comparing structures in three dimensions one can reduce computational burden by comparing a one-dimensional representation of structure, encoded as a “structural alphabet” (86). This approach was recently employed by van Kempen et al., who introduced the 3D interaction (3Di) structural alphabet and the Foldseek tool, which uses it to make efficient comparisons between structures (87). 3Di consists of 20 states, which correspond to the 20 letters also used for amino acid single-letter codes, but representing residue geometry relative to 3D neighbors rather than chemical identity. Foldseek’s efficiency, accuracy, and timely arrival shortly after large predicted-structure databases have made it a staple for navigating structural diversity. Practically similar to the NCBI BLAST web server, users can enter a query protein structure on the Foldseek web server (https://foldseek.com/) to retrieve structural homologs from a variety of databases of protein structures, including those from viruses. Although inexperienced users should take care when interpreting structural homology searches - paying particular attention to *e-value*, which relates to the likelihood of detecting a given homolog by chance (lower is better, 1×10^-3^ being a sensible maximum value cutoff), and *probability score* (ranging from 0-1, higher is better).

Beyond the web server, a local Foldseek installation permits custom exploration of homology. With this tool, we have gained early insights into the extent of protein novelty within the Virosphere. Independent comparisons of virus-focussed structure databases (e.g., Nomburg et al., BFVD, Viro3D, and Phold) with databases of cellular life (e.g. AFDB), demonstrate that >60% of viral protein folds have no detectable structural or sequence homologues in cellular life (8, 43, 67, 72). Moreover, even after sensitive structure-guided searches, a significant proportion of viral proteins remain “functionally dark”, confirming the Virosphere as a rich source of undiscovered biology (68).

With these tools and resources at our disposal, we now consider the questions we may ask of virus evolution, and the new perspectives structural homology can provide.

## 3 Taking a structural perspective on virus evolution

**Figure 2:**
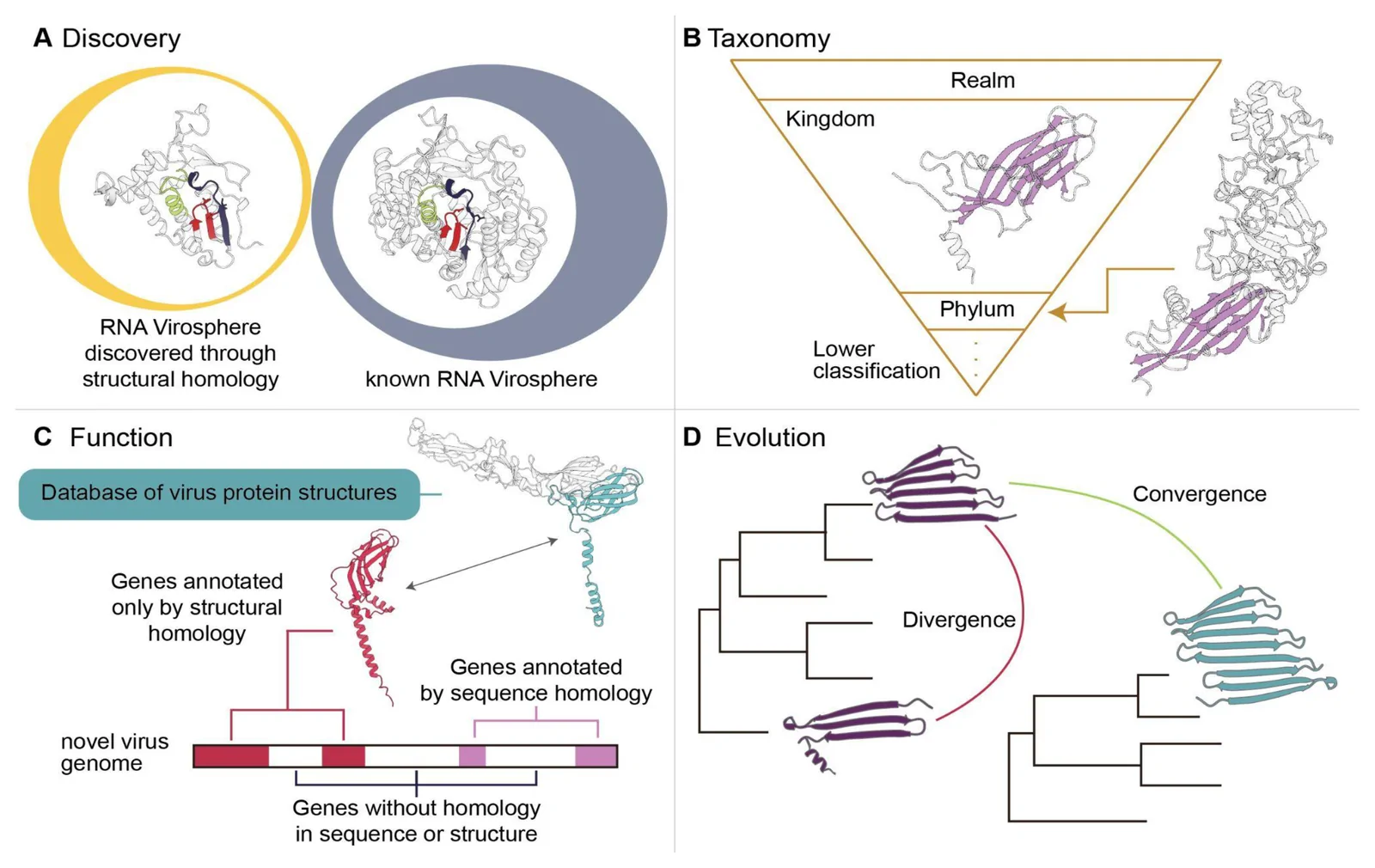
Insights on viral evolution provided by protein structure prediction. Key virus protein folds schematic and biological questions to answer. **A)** Comparison between the cryoEM structure of the poliovirus RdRP (1RA6, right) and the ColabFold predicted partial RdRP representative structure from RNA virus Supergroup 23 (left), detectable only through using the LucaProt method (94). Key conserved elements of the palm domain are annotated consistently with Edgar et al. fig. 2a (104). **B)** Illustration of the taxonomic placement of anelloviruses based on similarity between their capsid protein’s single jelly-roll (SJR) fold (8CYG, right) and the circovirus capsid protein (6RPK, left). The SJR fold is coloured in purple for both structures. **C)** Functional annotation of novel virus genomes based on sequence and structural homology. The example of structure-based annotation shown in the panel is between the metatranscriptomically discovered Maximus pesti-like virus polyprotein (red) and the Chikungunya virus E1 glycoprotein structure (6NK6, teal), also displayed in Petrone et al. fig. 1d (2). **D)** Divergent versus convergent evolution of repeating protein structures. The structure used for demonstrating convergence (teal, right) is the AFDB E1Y9I8 predicted structure, also displayed in Wright fig. 3 (139), while the other two are illustrative protein structures.

Viruses are not a single monophyletic group but rather a mosaic of lineages with independent origins, reflected in a megataxonomy divided into distinct realms based on hallmark genes. Indeed, the recognition of structural homology, particularly in capsid proteins (88), has been critical in defining this framework (89–91). While large-scale metagenomic surveys continue to reveal immense diversity of forms and lineages, likely with very ancient origins (92, 93), the ability to detect structural homology allows us to address questions that were virtually unattainable prior to the structure-prediction revolution (Figure 2).

First, structural searches can expand the known Virosphere, uncovering the viral identity of proteins previously classified as metagenomic ‘dark matter’ (94). This quest to characterise the fringes of the Virosphere can reveal divergent, potentially pathogenic viruses relevant to health (95–100) that remain undetectable by sequence alone. Second, structural relatedness can reconcile high-level virus taxonomy by identifying where ‘orphan’ taxa fit in the current system (101). The International Committee for the Taxonomy of Viruses (ICTV) has begun taking structural similarity into account when updating the taxonomy, as recently done for the *Flaviviridae* (102).

Third, structural conservation over long evolutionary timescales is driven by purifying selection to maintain biological function. Based on this principle, similarity to well-characterised folds allows us to infer the likely function of divergent sequences and map the distribution of mechanisms across the Virosphere (43, 103). Lastly, we can address a key question in protein evolution: whether complex folds evolved convergently or from a single origin. Comprehensive structural surveys across the tree of life make it possible to formally investigate this, while accounting for the frequent protein exchange between viruses and their hosts.

### 2.1 Virus discovery

**Figure 3:**
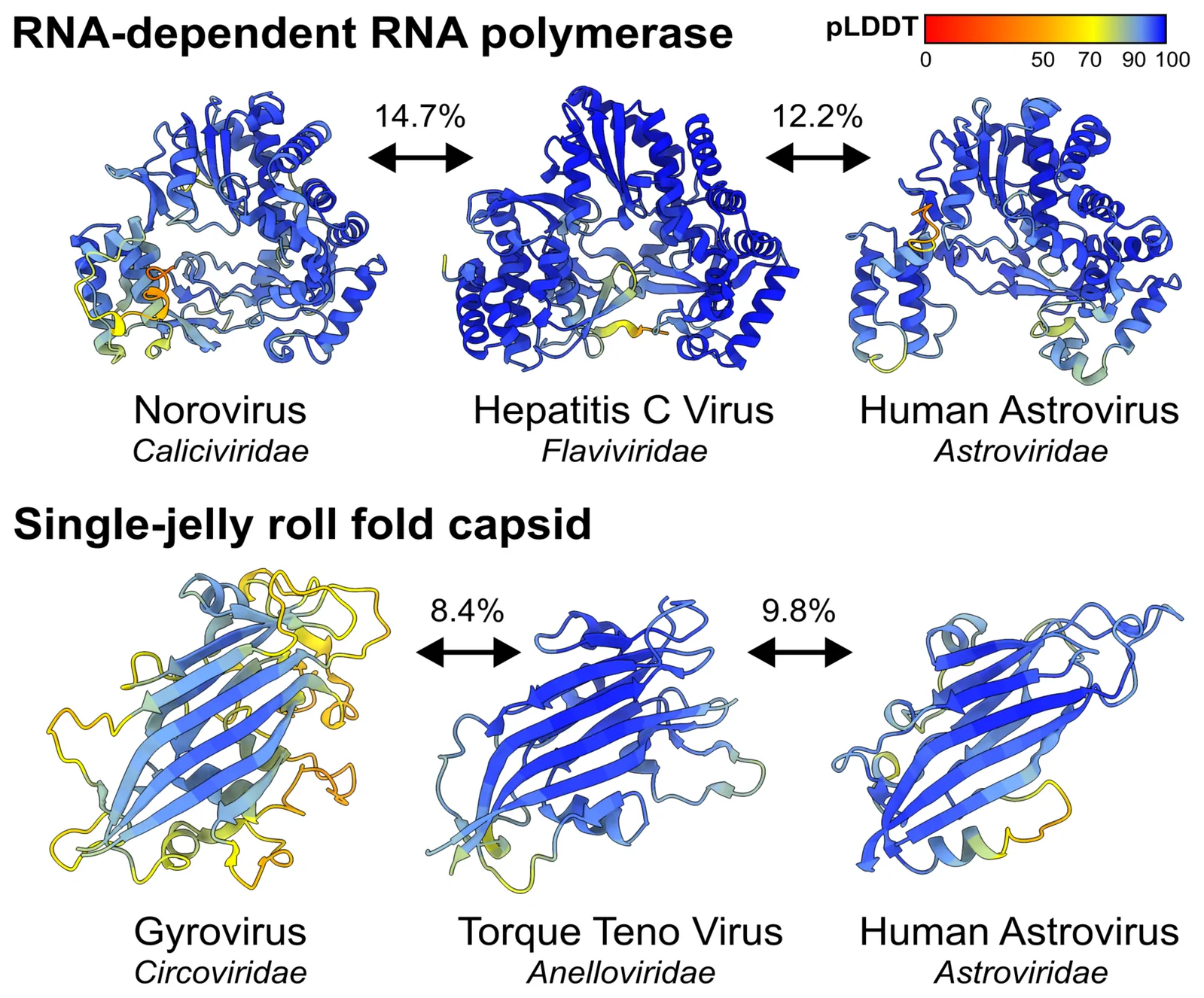
Conservation of structure, over sequence, in viral proteins. Example ColabFold predicted protein structures for RNA-dependent RNA polymerase (a hallmark protein of Riboviria) and single-jelly roll fold capsid protein (which is widely distributed across the Virosphere). In either case protein structure is highly conserved even when sequence conservation drops into the “twilight zone” (<25% amino acid identity). For each structure the virus common name and family are provided, with arrows and percentage values indicating amino acid sequence identity between neighbouring structures. All structures were taken from the Viro3D database (43) and are color-coded by pLDDT prediction confidence, as indicated by the key. Note that for clarity and ease of visualization, the inserted P-domain, described in the text for Anelloviridae (Torque Teno Virus), is not shown.

In RNA virus metagenomic discovery, the hallmark protein is the RNA-dependent RNA polymerase (RdRP) (Figure 2A). Beyond conserved sequence motifs, RdRPs preserve a unique structure across diverse lineages and taxa (Figure 3) (104–107). The catalytic core, known as the palm domain, typically consists of a three- or four-stranded beta-sheet flanked by alpha helices (108). This fold has an ancient origin, being related to the ubiquitous RNA-recognition motif (RRM) (109) and sharing structural homology with virus RdRPs, reverse transcriptases, and viral and cellular DNA-dependent DNA polymerases (110). Although the precise origin of this replication machinery remains elusive, the RRM likely represents the most ancient homologous protein shared by viruses and cellular life (111). Notably, cellular RdRPs possess a distinct double-psi beta-barrel core (112), indicating a separate evolutionary origin and functional convergence.

Recently, Hou et al. (94) surveyed >10,000 metatranscriptomes using their LucaProt method, which integrates sequence and predicted structure homology to discover novel RdRPs and, by extension, novel RNA viruses. The study identified 159 virus supergroups not classified by the ICTV, 60 of which were detectable only with LucaProt. This highlights the benefit of using high-throughput structure prediction over sequence-based similarity for diverse virus discovery. However, predicting the immense number of structures required for comprehensive searches remains technically challenging, even with pLM-based methods like ESMFold. Hence, recent developments in AI-based approaches have incorporated structural information into neural networks without predicting protein structure (53, 113). While the relative performance of these fast-evolving methods requires benchmarking, it is certain that unexplored parts of the RNA Virosphere remain to be discovered.

### 2.2 Reconciling the higher ranks of virus taxonomy

While focusing on the RdRP is useful for classifying most RNA viruses, some groups lack a polymerase and rely on host machinery for replication; for these, taxonomy is based on the capsid protein. However, unlike RdRPs, viral capsids exhibit immense structural diversity and likely have multiple origins, potentially acquired from hosts through many independent events across evolutionary time (90, 114, 115).

The *Anelloviridae*—a family of ssDNA viruses ubiquitous in humans and mammals—exemplify this challenge (116, 117). These viruses have short 2-4kb genomes characterised by large genetic diversity including frequent deletions and insertions (118, 119). Despite sharing a few features with the genus *Gyrovirus* (family *Circoviridae*), their high sequence divergence obscured any confident grouping, leaving *Anelloviridae* as the only eukaryotic circular ssDNA orphan taxon (120, 121).

Liou *et al*. (122) recently determined the cryoEM structure of the anellovirus capsid protein, revealing a single jellyroll (SJR) fold - the most prevalent fold among all known viral capsids, consisting of two four-stranded beta sheets facing each other (115, 123). Uniquely, however, long insertions in the loops between these sheets created a large ‘projection’ (P) domain in the anellovirus capsid. The hypervariable nature of the P domain and its interspersed placement within the SJR fold concealed sequence-based homology to other known virus capsids. By comparing predicted structures across the family, Boutkovic et al. (124) confirmed clear homology between the anellovirus and circovirus SJR folds (Figure 2B, Figure 3). Directly enabled by AI structure prediction, this work reclassified the ‘orphan’ *Anelloviridae* into the new phylum *Commensaviricota*, within the kingdom *Shotokuvirae* (realm Monodnaviria) (124).

Similarly, cryoEM has recently revealed the novel nucleocapsid architecture of baculoviruses. When combined with an AI-predicted structural “atlas” of conserved baculovirus proteins, this suggests they belong to a novel viral realm (125). These examples showcase the clear applicability of protein structure prediction for refining virus taxonomy, even in the presence of substantial structural diversification unique to specific virus taxa.

### 2.3 Functional annotation of diverse proteins

While taxonomy relies on hallmark proteins, such as polymerases or capsids, viruses encode many other proteins required for adaptation to their host or niche. As we trace evolutionary history, we observe frequent switching of these proteins between viral taxa and even domains of life. Hence, while non-hallmark proteins cannot be used for taxonomy their distribution amongst species offers critical insights into viral ecology and biology.

Metagenomic discovery frequently yields genomes that are classifiable by hallmark proteins but contain vast “dark” regions that are un-annotatable by sequence-based tools (e.g., BLAST, InterProScan) (126, 127) (Figure 2C). The inherent relationship between structural similarity and protein function can, however, be harnessed for sensitive protein detection and annotation. A notable example of this is members of the largely uncharacterised large-genome flaviviruses (LGF), which are diverse positive-sense ssRNA viruses primarily found in invertebrate metagenomic samples (128, 129). Whilst their RdRPs are homologous to those of orthoflaviviruses and pestiviruses (classified in the *Pestiviridae* family as of 2026) (102), LGF genomes are substantially longer, reaching 20 to 30kb, with divergent polyprotein regions matching no known proteins (24). The largest, a nearly 40kb genome from a sea sponge metatranscriptome, was analysed by Petrone *et al*. using structure prediction (2). This revealed divergent peptides with no sequence homology but detectable structural homology: a glycoprotein structurally similar to alphavirus E1 but sharing only 16% sequence identity; a Tu elongation factor-like structure (~10% sequence identity); and a divergent cassette of nucleic acid metabolism enzymes. Despite these insights, most of the polyprotein remained unannotated by any method.

Limitations in structure-based annotation stem primarily from prediction errors on divergent sequences and incomplete reference databases. Systematic efforts to overcome this have emerged in bacteriophage research. Bouras *et al*. (72) assembled a database of 1.36 million phage protein structures with functional labels, allowing their tool, Phold, to annotate divergent phage and archaeal virus genomes far beyond the capabilities of sequence-based approaches. Phold can automatically label nearly 50% of previously unannotated proteins (130). Its high throughput relies on a phage-specific ProstT5 language model that predicts 1D structural representations (3Di) directly from sequence, bypassing full 3D prediction, for comparison via Foldseek (53). Other recent methods, such as LucaVirus and ESM3, similarly employ multimodal language models trained on sequence, structure, and annotation to bypass computationally expensive folding steps (55, 113).

Alternatively, systematic comparison and clustering allow for protein-oriented searches - asking which species possess specific functions rather than annotating a given species. For example, Mifsud et al., deployed protein-specific Foldseek searches of a proteome-level structure set, to map the distribution of functions across the *Flaviviridae* (24). In doing so they revealed distinct evolutionary histories, even for proteins adjacent to one another in the viral polyprotein. For example, they showed that pestivirus E1 glycoprotein originates through genetic exchange with ancestral Hepaciviruses, whereas its polyprotein neighbour, E^rns^, arose through horizontal gene transfer of a bacterial RNase (24). Foldseek-based clustering of the Nomburg et al. dataset revealed major populations of hallmark proteins such as capsid proteins and RdRp, corroborating their use in taxonomic classification (8). In building Viro3D, Litvin et al. (43) went further by performing network analysis across structural clusters to efficiently discover many instances of a given fold. For example, mapping diverse class-I fusion glycoproteins revealed their distribution across multiple viral realms, including previously unknown instances such as in the *Herpesvirales* (43).

### 2.4 Divergence and convergence in protein structure

AI structure prediction has advanced the topics outlined above, yet one fundamental question remains largely unexplored: have complex structures evolved convergently throughout the history of life? The prevailing paradigm suggests that large, complex folds are unlikely to have evolved twice and, despite some being found across the tree of life, have originated from a common ancient source (131) (Figure 2D). This is supported by viral capsid diversity, in which several structurally dissimilar folds can assemble into identical virion architectures (123). If multiple distinct folds can form the same icosahedral shell, strong selection for convergence to a single fold (e.g., the SJR) is unlikely (115). Furthermore, common capsid folds appear in proteins with unrelated functions, suggesting that neither geometry nor function necessarily drives structural convergence (132).

Focusing on viral enzymes rather than ancient capsid proteins, we see similar patterns. Phosphodiesterases (PDE) which antagonise the hosts’ OAS-RNAse L antiviral response (133–135), are found in multiple RNA virus lineages, including members of the *Coronaviridae* (order *Nidovirales*), the *Torovirus* genus (family *Tobaniviridae*, order *Nidovirales*), multiple species of the *Rotavirus* genus (family *Sedoreoviridae*, order *Reovirales*) (8), and have structural homologues in DNA viruses, such as avian poxviruses (genus *Avipoxvirus*, family *Poxviridae*) and in bacteriophage (8, 136). Although most of these PDEs have very divergent sequences, Goldstein and Elde employed sequence and predicted structure comparisons to show that they share homology with the mammalian PDE family AKAP7 (137). This implies multiple independent acquisitions from hosts followed by diversification, rather than convergent evolution.

Although current evidence favors single origins for complex folds, there has been relatively little work exploring how often similar folds could evolve independently, or what the limits of such structural convergence might be. Databases of predicted protein structures encompassing all known parts of the tree of life are essential for testing such questions. By examining a clustered version of the AFDB (138), Wright (139) found that ~1% of AFDB structural homologue clusters showed no sequence-level homology (140). Notably, many of these were low-complexity proteins with repeated structural motifs, and tandem repeats on the sequence level. Similarly, Edgar and Sahakyan showed structural convergence in short motifs within otherwise unrelated proteins (141). This suggests that structural convergence is plausible for short, low complexity, repeat-containing architectures (Figure 2D). Conversely, large, complex structures are unlikely to have evolved multiple times convergently, even under selection for function, because acquiring an existing protein from another domain of life might be simpler and more parsimonious.

These hypotheses require further exploration, specifically within virology. Confidently predicting diverse virus proteins could help determine the threshold of protein complexity at which convergent evolution becomes unlikely, and whether the rapid evolutionary rate of viruses shifts this threshold. Ultimately, resolving these questions requires reconstructing coherent evolutionary histories; a task where protein structure is increasingly used to bridge vast evolutionary distances.

## 4 Integration of structure and phylogenetics

### 3.1 Structural phylogenetics for deep evolutionary inference

Beyond pairwise homology searches, large structural databases permit systematic comparisons within groups of homologous proteins to disentangle deep evolutionary histories. Sequence-based phylogenies are fundamentally limited when amino acid identity falls into the “twilight zone” (<25%), where alignments become unreliable and phylogenetic signal degrades (15, 25). Furthermore, hallmark genes under strong purifying selection can also exhibit saturation over deep timescales, obscuring true divergence patterns (142) and producing apparent “evolutionary stasis” (143, 144). This reflects a mutation-selection balance operating under highly constrained amino acid site preferences (145): most positions in these proteins can only accommodate a small subset of residues without disrupting folding or function. Once these preferred states are fixed, substitutions become rare and the apparent evolutionary rate declines, even though mutation pressure persists (23).

Protein structure offers an additional layer of information for resolving deep evolutionary relationships. Incorporating structure into phylogenetic inference can extend ancestral reconstructions much further back in time and recover relationships that are otherwise inaccessible to conventional approaches (24, 25). Early methods reconstructed distance trees based on metrics describing the similarity of superposed structures (e.g., via TM-Score). However, these methods were limited by pitfalls also encountered in sequence-based distance approaches, such as poor scaling to larger and more diverse datasets and discrepancies depending on the distance metric used (146, 147). Structural phylogenetics methods using atomic geometry are comprehensively reviewed in Puente-Lelievre et al. (148).

With the development of Foldseek, structural alphabet-based methods have gained substantial popularity (87). The 3Di alphabet is largely compatible with existing methods for amino acid sequence alignment (149) by replacing the substitution model with an empirical 3Di matrix (25, 150). Recently, Gilchrist et al. developed FoldMason (151), a tool that uses both 3Di structural homology and amino acid homology to construct structure-informed MSAs. Moreover, 3Di character MSAs can be used in conventional phylogenetic frameworks including neighbor-joining (e.g., FoldTree (152)) and maximum-likelihood tools such as IQ-TREE (153), which can infer on partitioned models of both sequence and structural similarity (24, 25). Implementation of these methods is becoming increasingly convenient with the development of computational pipelines streamlining structural phylogenetic workflows (e.g. Unicore (154)).

Integrating structure into phylogenetics, however, introduces significant challenges. Unlike sequences, structure is a phenotype - a product of both the genome and the physicochemical environment. Consequently, structural characters (like 3Di states) are inherently non-independent; changes in one region of a protein can induce coordinated adjustments elsewhere which will affect local structure and, correspondingly, the 3Di states of neighbouring residues. While amino acid sites exhibit epistasis, interdependence is potentially stronger for structural features, complicating standard phylogenetic assumptions of site independence. Reliance on predicted structures adds another complexity due to the co-evolutionary signals used in MSA-dependent prediction.

Interpreting branch lengths in structural phylogenies remains another open question. In sequence trees, lengths represent substitutions per site; in structural trees, they reflect changes in the protein’s underlying geometry or fold topology of the protein. A transition from one 3Di state to another is a “substitution” in representation, yet the magnitude and nature of the underlying conformational change can vary widely. Moreover, coordinated conformational shifts can alter many 3Di states without any accompanying sequence changes. Developing appropriate substitution models for structural phylogenies is an active area of research (150). Addressing these challenges is essential for unifying sequence and structural evidence into a coherent framework for exploring deep viral evolution. Although Mutti et al. show that structural phylogenetics do not outperform sequence-based methods for reconstructing eukaryotic diversity (155), we expect that these methods will substantially improve the reconstruction of relationships between the much more divergent proteins of viruses (24, 43).

### 3.2 Comparing sequence and structural divergence

**Figure 4:**
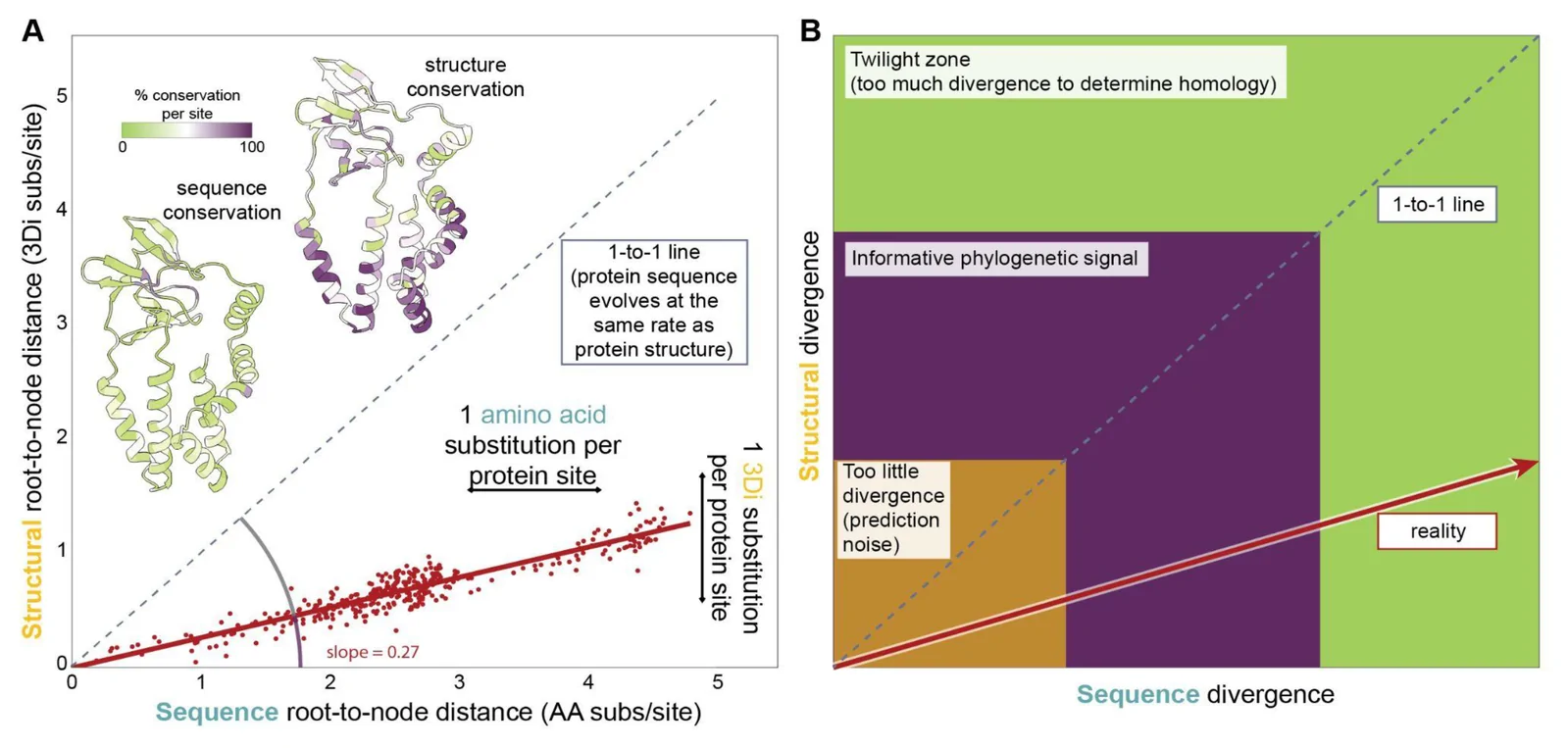
Comparison of virus protein sequence and structural divergence. **A)** Scatter plot and linear regression between amino acid (x axis) and 3Di (y axis) substitutions on each branch of the E1 glycoprotein Hepaciviridae and Pestiviridae phylogeny inferred using a partitioned amino acid and 3Di phylogenetic inference by Mifsud et al. (24). The one-to-one trend, where sequence evolves at the same rate as structure, is shown as a dashed line for comparison. On the top left of the panel the ColabFold-predicted hepatitis C virus E1 glycoprotein structure (168) is presented, coloured by amino acid (left) and 3Di (right) per residue conservation across the E1 proteins used for the tree reconstruction. Both structures are coloured on the same scale of percentage conservation. **B)** Illustrative schematic of the expected relationship between sequence and structural divergence. The shading of the plot corresponds to the amount of phylogenetic information available at each point in sequence or structural protein divergence.

A direct comparison of amino acid and 3Di substitution accumulation rates can be made for the E1 glycoprotein structure-guided phylogeny presented in Mifsud et al. (24). E1 is structurally conserved across the Pestiviridae and Hepaciviridae but exhibits high sequence divergence (102). In Figure 4A, we have constrained the tree topology (constructed using a partitioned amino acid and 3Di model) and reinferred the tree’s branch lengths first consisting of amino acid characters (sequence, x-axis) and second of 3Di states (structure, y-axis). Surprisingly, we observe an approximately linear relationship, likely reflecting the fact that both sets of branch lengths were inferred on the same fixed tree topology under their respective substitution models. However, structural substitutions accumulate at roughly one-quarter of the rate of amino acid substitutions (Figure 4A). This aligns with Illergård et al.’s estimate that structural features evolve three to ten times more slowly than sequence (156). Note that the 3Di-based branch lengths used here and the RMSD-based distances used by Illergård et al. quantify structural change differently, but both are intended to capture structural divergence.

Mapping sequence and structural conservation onto the E1 protein reveals spatial heterogeneity (Figure 4A). The 3Di states of the transmembrane helix residues are largely invariant, whereas exposed regions show significant structural variability. Conversely, the amino acid sequence is highly variable throughout, with only sparse conservation in exposed regions. This underscores the need for models that account for site-specific rate variation in structural phylogenetics, analogous to standard treatments of among-site rate heterogeneity in amino acid models (157, 158).

These observations suggest a framework in which the slower divergence of structure allows it to capture a different window of phylogenetic signal in the timescale of protein evolution and sequence divergence (Figure 4B). Even when amino acid divergence has entered the “twilight zone”, structural characters retain sufficient signal to reconstruct evolutionary relationships. Conversely, when divergence is low, structural differences provide limited resolution, making sequence data (amino acid or even nucleotide) more reliable. Noise and inaccuracy in predicted or experimental structures exacerbates this, as small, uncertain differences between similar protein structures may conflate phylogenetic reconstruction (Figure 4B). Thus, structural characters, like 3Di states, function as an “effective slow clock” - continuing to tick after conventional clocks saturate, but still requiring a minimum threshold of divergence to become informative.

### 3.3 Promises and pitfalls of structural clocks

The motivation for moving beyond sequences is straightforward: purifying selection and genomic site saturation cause sequenced-based clocks to “freeze” or “slow down” over deep timescales. In rapidly evolving RNA viruses, multiple hits at constrained sites result in an underestimation of divergence, giving rise to pronounced time-dependent clock-rate behaviour. The Prisoner-of-War (PoW) framework (144) formalises this: viral genomes explore a restricted sequence space in which rapidly evolving sites saturate first and more constrained sites dominate at longer timescales. Protein structure provides a complementary, slower-evolving readout of these deep relationships, but treating it as a clock-like trait poses specific challenges.

First, the coarse-graining that makes 3Di attractive for deep time also creates fundamental complications for defining and estimating an “evolutionary rate of structure”. Grag and Hochberg emphasize that 3Di compresses many distinct amino acid configurations and coordinate arrangements onto the same 3Di state (150). While this overcomes saturation, it sacrifices resolution at short timescales. In PoW terms, 3Di behaves like a “binned” sequence space: able to recover broad trends after fine-scale variation is erased, but insufficiently granular to resolve tip-level substitutions. Consequently, 3Di branch lengths near tips are hard to interpret as simple counts of change. Furthermore the added value of 3Di varies with structural complexity; Fullmer et al. showed that additional resolution is low for short alpha-helical proteins (159).

Second, structural evolution is highly heterogeneous and non-local. 3Di characters reflect the relative arrangement of a residue and its neighbours in 3D. Thus, an insertion that introduces a new tertiary element can change the 3Di state of many surrounding positions without any local amino acid replacement (150). In the context of an alignment this looks like a burst of rapid evolution, despite sequence change being sparse and highly localised. Such dependencies violate standard time-reversible Markov models that assume independent sites. Co-evolution and epistasis, which are well-recognized at the sequence level (160), are even more pronounced in structure, potentially inflating the apparent rate variation across branches and making structural data look less clock-like.

Third, conformational dynamics and fold-switching introduce ambiguity. Proteins occupy ensembles of conformations, and in some cases undergo bona fide fold switches as part of their function. Garg and Hochberg demonstrate that if a sequence is modelled in two distinct conformations (e.g., ground vs fold-switched KaiB), 3Di sequences cluster by conformation rather than genealogy - essentially reconstructing a phylogeny of states rather than a phylogeny of sequences (150). In viral glycoproteins and polymerases, where temperature, ligand binding or membrane environment can induce large conformational shifts, similar issues will arise if a single static model is treated as a faithful representation of “the” structure. Structural clocks based on one conformation risk conflating genuine evolutionary divergence with differences in which part of the conformational landscape has been sampled or predicted (161–163). This is exacerbated by AI prediction methods, which typically output a single conformer for a given sequence and its close relatives, but may output alternative, but functionally valid, conformations for more divergent instances (164).

These issues fuel questions of whether protein structures obey a molecular clock. Recent work appears contradictory. Pascual-García et al. (165), found more frequent clock violations in structure than in sequence using atomic coordinate-based distances, whereas Moi et al. (152) reported that 3Di sequences are more ultrametric than amino acids, showing a tighter spread of root-to-tip distances and thus appearing more clock-like. ￼Puente-Lelievre et al. (25) argue that both results can be true: “clockliness” is not an intrinsic property of the protein but of how its 3D structure is represented and quantified. ￼Atomic coordinates are sensitive to conformational noise, making functional shifts look like large, irregular jumps. In contrast, 3Di collapses structural fluctuations into the same state, smoothing this punctuated behaviour and yielding more uniform path lengths, but this comes at the cost of discarding fine-grained geometric detail, so distinct conformational or evolutionary changes may be mapped onto the same 3Di states and therefore appear artificially similar.

For deep viral evolution, a cautious strategy is required. Structural characters clearly extend the usable window of divergence, and 3Di-based analyses show robustness to long-branch attraction and saturation in highly divergent viral families (24, 43). However, given purifying selection, epistasis, and PoW-style saturation, neither sequence nor structure will behave as a reliable clock across tens or hundreds of millions of years. A pragmatic near-term approach is to treat structural data as a complementary, slower-evolving partition rather than a wholesale replacement for sequences: joint amino-acid + 3Di models with separate substitution processes and (ideally) separate branch-length scalings can capture the fact that “one unit of time” does not correspond to the same number of changes in each representation. In the longer term, “structural clocks” for viruses must be mechanistically explicit, embedding PoW-like saturation at the sequence level within models that describe how selection on fold stability, conformational flexibility, and interface geometry translates into rare, often punctuated changes in structural alphabets. Such models will be challenging to formulate and fit, but they offer a principled route to converting structural similarity into more meaningful estimates of evolutionary distance and timescale.

## 5 Future Directions

With their complex, entangled origins, and rapid evolution, viruses pose a formidable challenge for any approach to studying deep evolutionary history. As the integration of AI-structure prediction into homology searches, taxonomic classification, and phylogenetics continues to mature, we may expect investigations of viruses to sit at the frontier of what is possible.

Looking forward, utility will depend on specific technical advances. For structure-guided searches, increased performance on divergent, MSA-poor sequences would be beneficial; improvements here would not only illuminate extant “functionally dark” sequences - such as those of the Large Genome Flaviviruses - but also drive new waves of discovery at the fringes of the Virosphere, building on the success of approaches like LucaProt. Furthermore, accurate conformational sampling of fold-switching proteins would provide control over the inputs to homology searches, mitigating noise from dynamic proteins. Recently, the greatest progress has been made in predicting protein-protein interactions (PPI) (166). While monomeric structure resolves deep homology, the ability to model complexes offers a window on the drivers of viral evolution. Consequently, high-throughput PPI prediction may allow us to map the “selective landscape” that constrains and directs viral evolution over deep timescales.

However, caution is required over the provenance of these tools. Foundational AI models are typically developed outside of the virology community. In some recent instances, such as the ESM3 pLM, viral data have been deliberately down-weighted or excluded from training to mitigate against dual-use pathogen design (55). Therefore, new and emerging tools require careful and systematic benchmarking against viral target sequences to safeguard the delivery of high-quality viral structure datasets.

Nonetheless, performance at CASP16 suggests that, for most targets, the structure prediction problem is nearly solved (167); thus, there are few technical barriers to systematic structure-guided exploration of viral evolution. Indeed, the greatest unmet need is a single harmonised reference database that adequately covers both sequence and structural diversity. This will require classifying and annotating exemplar sequences across known viral diversity, as many shortcomings of existing virus structure databases stem from incomplete or fragmentary sequence annotations. This process will be expedited by the integration of structure-informed annotation tools, but will necessitate collaboration and coordination across the community. Thankfully, as of February 2026, the AFDB is open to community datasets including large-scale virus-focussed databases, and both Viro3D and BFVD were included in the first data integration. We may expect this to catalyse concerted and unified efforts to assemble a definitive “one-stop” dataset for structure-guided exploration of virus evolution, and how this intersects with cellular life.

Finally, a fundamental limitation remains; we are ultimately restricted to studying extant viruses. While the combination of massive metagenomic sampling, discovery of new endogenous viral elements, and protein structure prediction will illuminate deeper evolutionary timelines, the turnover and extinction of viral lineages mean that stretches of evolutionary history will have been erased. Consequently, despite the resolving power of structure-informed phylogenetics, some gaps in the origins of viral species and proteins may remain unbridgeable. Nonetheless, there is much to be learned before we reach this final frontier.

## Figures and Tables

**Table 1:** Available databases of predicted virus proteins, Spring 2026.

Database	Focus/Sequence Set	Method	Website/Data Download
**Nomburg et al. 2024** (8)	Representative NCBI RefSeq eukaryotic viruses (4463 genomes, 67,715 entries).	ColabFold (33, 35)	https://zenodo.org/records/10291581
**Big Fantastic Virus Database (BFVD;** (67))	Prioritised coverage of structural diversity using representatives of the UniRef30 clusters (351,242 entries).	ColabFold (33, 35)	https://bfvd.foldseek.com/
**Viro3D** (43)	Proteome-level coverage of ICTV ratified human and animal viruses (4407 genomes, 85,162 entries).	ColabFold, ESMFold (33, 35, 52)	https://viro3d.cvr.gla.ac.uk/
**Viral AlphaFold Database (VAD;** (68))	Prioritised coverage of structural diversity using representatives of NCBI RefSeq clustered at 30% sequence identity. Performed monomer and dimer predictions (26,962 entries).	AlphaFold2 (33)	https://vad.atkinson-lab.com/
**Phold** (72)	Comprehensive coverage of bacteriophage. Clustering phage proteins from the PHROG, enVhog, efam, DGR, ACR, CARD, DefenseFinder, VFDB, and NetFlax databases (3,160,000 entries).	ColabFold, ESMFold (33, 35, 52)	https://github.com/gbouras13/phold
**MetaVR** (69)	Representative sequences of MetaVR protein clusters with at least 15 unique members (748,927 entries).	AlphaFold3 (34)	https://www.meta-virome.org/
**Mutz et al. 2025** (70)	Annotated and unannotated ORFs of nearly 500 operationally defined virus families (24,624 entries).	ColabFold, ESMFold (33, 35, 52)	https://zenodo.org/records/13915592
**RNA Viral Protein Structure Database** (71)	RNA virus protein sequences, drawn from ICTV ratified species and GenBank *Riboviria* entries (5,263 genomes, 154,716 entries)	AlphaFold2 (33)	https://virus.9itsg.net/#/home

## List of abbreviations

3Di: 3D interaction
AFDB: AlphaFold Protein Structure Database
AI: Artificial Intelligence
AKAP7: A-kinase anchor protein 7
BFVD: Big Fantastic Virus Database
BLAST: Basic Local Alignment Search Tool
CASP: Critical Assessment of Structure Prediction
cryoEM: Cryo-electron microscopy
ESM: Evolutionary Scale Modeling
ICTV: International Committee for the Taxonomy of Virus
LGF: Large Genome Flaviviruses
MSA: Multiple Sequence Alignment
OAS: 2′-5′-oligoadenylate synthetase
PDB: Protein Data Bank
PDE: phosphodiesterase
pLDDT: predicted Local Distance Difference Test
pLM: protein Language Model
PoW: Prisoner-of-War
PPI: protein-protein interactions
pTM: predicted template modelling
RdRP: RNA-dependent RNA polymerase
RRM: RNA-recognition motif
SJR: single jelly-roll
VAD: Viral AlphaFold Database
